# PlasmaBlade-assisted surgical septal myectomy: technique and our experience

**DOI:** 10.3389/fcvm.2024.1345540

**Published:** 2024-01-31

**Authors:** Pankaj Garg, Amy Lykins, Mohammad Alomari, Jordan P. Reynolds, Elizabeth Johnson, Basar Sareyyupoglu

**Affiliations:** ^1^ Department of Cardiothoracic Surgery, Mayo Clinic, Jacksonville, FL, United States; ^2^ Department of Laboratory Medicine and Pathology, Mayo Clinic, Jacksonville, FL, United States; ^3^ Department of Radiology, Mayo Clinic, Jacksonville, FL, United States

**Keywords:** hypertrophic obstructive cardiomyopathy, septal myectomy, left ventricular outflow tract obstruction, PlasmaBlade, systolic anterior motion (SAM)

## Abstract

**Background:**

The pulsed-electron avalanche knife (PEAK) PlasmaBlade provides an atraumatic, scalpel-like cutting precision and electrocautery-like hemostasis. PlasmaBlade operates near body temperature, and its long, thin, and malleable tip can overcome the limitations of a surgical knife. In this study, we aimed to evaluate our clinical experience and histopathological outcomes of septal myectomy using PlasmaBlade.

**Methods:**

Electronic medical records were reviewed for preoperative, operative, and follow-up data of the patients who underwent septal myectomy using PEAK PlasmaBlade at our institute between January 2019 and December 2022. Histopathology of the myectomy specimens was reviewed for the depth of muscle necrosis and compared with the left atrial appendage (LAA) specimen.

**Results:**

Twenty-nine patients underwent septal myectomy using the PEAK PlasmaBlade. No mortality was reported. The mean age was 60.6 ± 12.5 years, and 58.6% of patients were male. Peak left ventricular outflow tract (LVOT) gradients were 40.5 ± 34.9 mmHg at rest and 56.5 ± 34.9 mmHg after provocation. Concomitant procedures performed were LAA ligation in 20 (69.0%), aortic valve replacement in 5 (17.2%), and coronary artery bypass grafting in 3 (10.3%) patients. Postoperative complications were complete heart block in one (3.4%) and ventricular septal defect in two (6.9%) patients. Both the ventricular septal defects were identified intraoperatively and repaired. Histopathology of myectomy specimens demonstrated cautery artifact limited to <50 µm depth compared to >1,000 µm with conventional electrocautery. At a mean follow-up of 8.4 ± 10.3 months, the mean LVOT gradient was 4.4 ± 5.8 mmHg at rest and 9.5 ± 3.3 mmHg after provocation. All patients were alive and in New York Heart Association class I/II. No patient developed complications or required reintervention or reoperation.

**Conclusion:**

Adequate septal myectomy can be precisely and safely performed using the PEAK PlasmaBlade with minimal collateral damage.

## Introduction

Surgical septal myectomy (SSM) is the preferred treatment for the relief of dynamic left ventricular outflow tract obstruction (LVOTO) in patients with hypertrophic obstructive cardiomyopathy (HCM) who remain symptomatic despite maximum tolerated medical therapy. Operative mortality of surgical septal myectomy is <1% in experienced centers (1), and long-term survival is 94% at 5 years and 91% at 10 years. Potential complications of septal myectomy include complete heart block (CHB), ventricular septal defect (VSD), and coronary-cameral fistula (1–4). Septal resection is conventionally performed using a surgical knife and sharp scissors. The main advantage of a surgical knife is its precision and sharp cut (5), while there is a small risk of inadvertent injury to the aortic or mitral valve (2, 6).

The pulsed-electron avalanche knife (PEAK) PlasmaBlade is an electrosurgical device that provides atraumatic, scalpel-like cutting precision and electrocautery-like hemostasis. Primarily, the PlasmaBlade uses pulsed radiofrequency energy to generate a plasma-mediated discharge along the exposed rim of an insulated blade. Plasma is an electrically conductive cloud created when the energy contacts tissue. The “plasma” allows the radiofrequency energy to cross the tissue at a much lower overall power level, resulting in lower operating temperatures and less thermal damage (7). The PlasmaBlade operates at 40–100°C, while the blade tip stays near body temperature. Therefore, underlying tissue damage is limited. Studies reporting the use of the PlasmaBlade during internal mammary artery harvesting or ocular surgeries have found the area of collateral damage to be only 2–10 μm with the PlasmaBlade compared to 100–400 μm with electrosurgery (7, 8).

The PlasmaBlade has been extensively used in ophthalmologic, plastic, and dermatological surgeries as precisely as a scalpel with the hemostatic control of conventional electrosurgery. Studies have also reported a reduced risk of bleeding, tissue injury, and scar formation with the PlasmaBlade (9–11). However, experience in cardiac surgery is limited to pocket creation for the pacemaker and implantable cardioverter defibrillator (ICD) implantation (12, 13) and internal mammary artery harvesting (7).

In our institute, we have used the PlasmaBlade to perform SSM for the last 4 years. The purpose of this retrospective study is to review our clinical outcomes and the effect of PlasmaBlade cutting on the excised muscle in terms of necrosis and inflammation.

## Methods

The study was approved by the ethical committee of Mayo Clinic (IRB number: 21-011885), and consent from the patients was waived, given the study's nature. Between January 2019 and December 2022, 29 patients underwent surgical septal myectomy using the PlasmaBlade for symptomatic dynamic LVOTO or as part of other cardiac surgical procedures.

Data were collected from electronic medical records. Transthoracic echocardiographic (TTE) data were collected according to the American Society of Echocardiography recommendations. The following echocardiographic parameters were abstracted: resting and provoked LVOT and mid-ventricular maximal instantaneous gradient, interventricular septal thickness, systolic anterior motion of the mitral valve (SAM), mitral regurgitation (MR), and LV ejection fraction. In postoperative echocardiography, additional data were logged for the presence of VSD and coronary-cameral fistula in addition to the above parameters. Cardiac contrast-enhanced computed tomography (CECT) and cardiac magnetic resonance imaging (CMRI) were also reviewed for any additional findings. For patients who underwent coronary angiography, data were recorded for the presence of coronary artery disease (CAD).

### Histological investigations

Four samples of 5 μm sections were cut and analyzed. Two samples were taken from the myectomy specimens, and two were taken from the left atrial appendage (LAA) of the cardiectomy specimens from the patients undergoing heart transplantation. Conventional electrosurgical cautery is a common histologic artifact found in these cardiectomy specimens since, at our institute, we routinely use conventional electrocautery to excise the LAA and prepare the recipient's LA cuff during heart transplantation to achieve hemostasis and prevent postoperative bleeding from the raw surface. Routine staining with hematoxylin and eosin (H&E) was performed. Evaluation of the PlasmaBlade and cautery artifact was performed by a single pathologist at 100×, 200×, and 400× magnification. Digital imaging scanning was performed using a GT450 light microscopy scanner for precise myocardial thermal artifact measurements. To prevent observer bias, the pathologist was blinded about the hearting or electrical element used on the myectomy specimen.

### Cardiac computed tomography/magnetic resonance imaging

Postoperative cardiac CECT (GE Healthcare, GE Discovery 750 HD) was reviewed in two patients for residual LVOT obstruction. Both these patients underwent cardiac CECT during the follow-up for anatomical modeling.

### Operative procedure for transaortic septal myectomy

Prior to initiation of cardiopulmonary bypass (CPB), intraoperative transesophageal echocardiography (TEE) was performed to re-assess the extent and distribution of ventricular septal thickening, SAM, mitral valve morphology, subvalvular apparatus, degree and direction of MR, and LVOT gradient at rest. All patients underwent resting and provoked LVOT gradient measurement by recording pressure from the ascending aorta and LV needle catheterization (24-Fr spinal needle) before and after myectomy. Electromechanical premature ventricular contraction (PVC) was induced to measure provoked LVOT gradients. Based on preoperative echocardiography, CECT chest, and/or CMRI, supplemented with intraoperative TEE findings, the transaortic vs. transapical vs. combined approach was decided. As suggested by Nguyen and Schaff, we selected the transaortic approach in all the patients with basal and mid-cavity obstruction, while the transapical approach was used for patients with apical hypertrophy. In patients with complex mid-cavity obstruction deemed difficult to relieve completely through the transaortic approach alone, an additional apical incision was made to completely relieve the obstruction (14).

**Figure 1:**
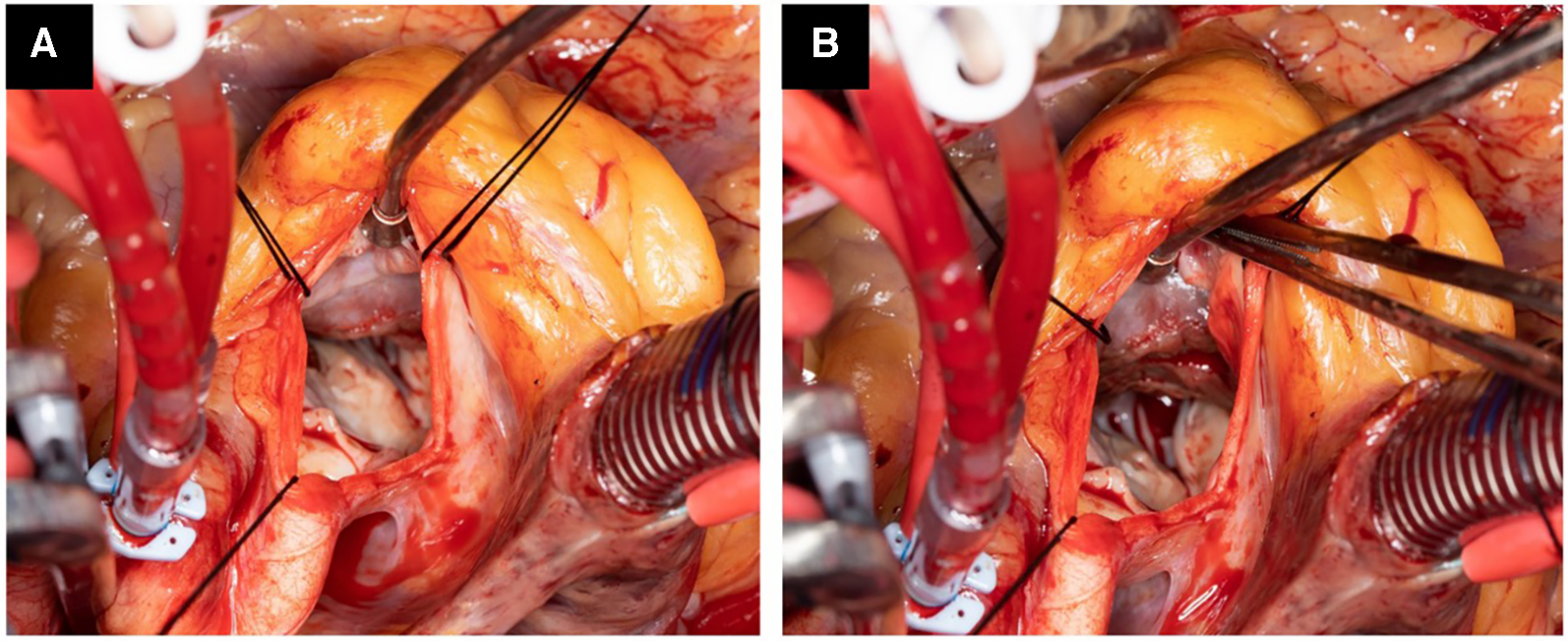
Operative photograph showing hypertrophied septum with scar tissue starting 1.5 cm below the aortic valve with significant narrowing of the left ventricle outflow tract (**A**). After septal myectomy, the left ventricle tract is wide open (**B**).

**Figure 2:**
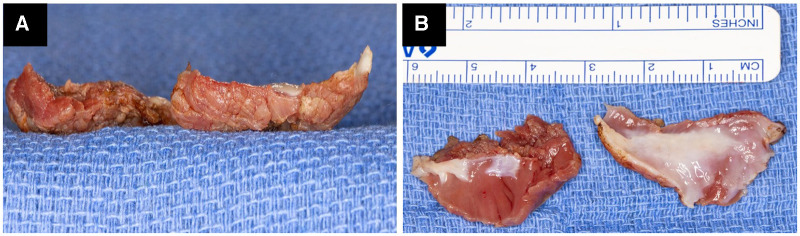
Operative photograph of two resected myectomy specimens showing the absence of charring and minimal electrocautery artifact on the raw surface (**A**). Both myectomy specimens are almost 3 cm × 2 cm × 0.7 cm in size, with the presence of scar tissue and almost complete absence of electrocautery artifact (**B**).

CPB was established by cannulating the ascending aorta and right atrium or superior and inferior vena cava, depending upon the additional surgical procedure required. After cross-clamping the aorta and arresting the heart, a transverse aortotomy was created, and stay sutures were placed to retract and keep the aortotomy open (Figure 1). The extent of the scar on the interventricular septum, which is the contact lesion produced by the apposition of the septum and the anterior mitral valve leaflet, was observed carefully. This scar serves as a guide to the length of the myectomy, which must be extended apically beyond the contact lesion. We initiated ventricular muscle resection, typically 0.5–0.75 cm below the nadir of the right coronary cusp and the commissure between the left and right cusps, and the resection was extended distally between these two lines down to the mid-ventricle. With the PlasmaBlade, a horizontal plane was created by bending the tip of the PlasmaBlade at a 45° angle, and muscle tissue penetration was performed at the desired depth (usually 8–10 mm). While the penetrated muscle was retracted, we continued the horizontal cut and maintained the thickness while advancing to the LV apex. Using the PlasmaBlade, we can easily remove the large piece of hypertrophied septum without any significant charring or necrosis of the septal muscle or collateral damage to the underlying conduction bundle (Figure 2). Once the desired distance reached from the aortic valve annulus, we repeated this horizontal shaving approach until we were satisfied with the remaining septal thickness. We paid diligent attention to the complete excision of the septal scar. We kept the septal intervention depth more conservative under the membranous septum to avoid injury to the His bundle and prevent CHB. If required, we also used a sponge stick to push the interventricular septum to improve the visibility of the mid-septum as described by Schaff et al. (15). The extent of muscular resection was dependent on the distribution and thickness of the basal septum, septal scar created by SAM, and extension of the septum from anterolateral to posteromedial commissures of the mitral valve. We believe that adequate extension of myectomy incision toward the LV apex is critically important as incomplete muscle resection can lead to residual SAM and LVOT gradient. In our experience, an extension of septal excision medially (clockwise) is unnecessary and may increase the risk of CHB. Abnormal papillary muscles inserting into the body of the anterior leaflet of the mitral valve may potentially crowd the LVOT, contributing to obstruction, and were excised, while anomalous muscles inserting into the free edge of the anterior mitral leaflet are unlikely to contribute to LVOT obstruction and were preserved. Similarly, chordal attachments from an anomalous papillary muscle to the free edge of the anterior mitral leaflet were not resected. In such conditions, septal myectomy was extended beyond the contact point of the papillary muscle and ventricular septum, and adjunctive surgical debulking of the papillary muscle was done to eliminate the potential for mid-ventricular obstruction. If the scar was present distal to the papillary muscles or difficult to approach through the aorta, an additional apical incision was made, and the rest of the resection was completed. Mitral ring annuloplasty was performed in one patient with severe MR judged to be caused by intrinsic mitral valve pathology. After adequate resection, aortic and mitral valves were inspected for any inadvertent injury, and aortotomy was repaired in two layers with a 4-0 polypropylene suture.

### Surgical technique of transapical septal myectomy

In patients who required apical myectomy as an isolated or additional procedure, the apex of the heart is elevated anteriorly into view using 2–3 moist laparotomy pads, and an incision, 4–5 cm in length and 1–1.5 cm away from the left anterior descending coronary artery, was made with a No. 10 blade on the LV apex. This incision was guided by LV apex palpation for the thin area of apical ballooning. Using the PlasmaBlade, the septal muscle was excised from the mid-ventricle and progressed proximally to join the subaortic resection. This excision was guided by the areas of endocardial scarring. Following adequate resection to relieve obstruction at the mid-ventricle, the ventriculotomy was closed linearly in two layers with 2-0 polypropylene sutures over a felt strip buttress.

Subsequently, the aortic cross-clamp was removed, and patients were weaned of CPB after the resumption of sinus rhythm with good contractility or after initiating the atrioventricular sequential pacing through temporary epicardial pacing wires in patients with complete heart block or through a pre-existing permanent pacemaker. After the patients were separated from CPB, postoperative TEE was performed to assess LVOT resection adequacy and the presence of MR, SAM, VSD, or coronary-cameral fistula. Resting and provoked LVOT gradient was measured again on TEE to confirm the complete relief of LVOT obstruction and resolution of MR. For a visual understanding of the technique, a detailed operative video of our technique has recently been published in the *Journal of Thoracic and Cardiovascular Surgery—Techniques* (16).

### Additional procedures

To decrease the risk of postoperative embolic stroke, the LAA was excised and closed, clipped with an AtriCure clip, or stapled in 20 patients. The data available in the literature are mixed about the role of prophylactic LAA ligation in decreasing the incidence of postoperative stroke (17, 18). If possible, we, at our institution, routinely perform LAA ligation in all the patients undergoing cardiac surgery on CPB. In two patients with a history of atrial fibrillation, pulmonary vein isolation was carried out. Mitral valve repair or replacement was performed only if patients had additional mechanisms of mitral regurgitation apart from SAM. We performed the mitral valve intervention at the time of initial septal myectomy if there was evidence of intrinsic mitral valve disease or dilated mitral annulus. Otherwise, we re-assessed the mitral valve on TEE after weaning from CPB and optimizing the hemodynamics with volume, vasopressors, and inotropes. If the patients had more than mild mitral valve regurgitation, we re-commenced the CPB and performed the mitral valve intervention on mild hypothermic cardioplegic arrest. For the mitral valve intervention, the valve was approached through Waterston's groove. The mechanism of mitral valve regurgitation was assessed using a saline test (19, 20). Mitral valve repair was preferred whenever possible. If mitral valve repair was deemed complex or inadequate, mitral valve replacement was performed. For mitral valve repair, ring annuloplasty with or without other mitral valve procedures was performed, while mitral valve replacement was performed with a mechanical or a bioprosthetic valve, depending upon the age of the patient and the patient’s wish. Other additional procedures performed were mitral valve repair or replacement, aortic valve replacement (AVR), coronary artery bypass grafting (CABG), left anterior descending coronary artery unbridging, LVOT mass excision, and excision of aortic valve fibroelastoma.

### Postoperative management and follow-up

Following surgery, high afterload was maintained using vasopressors to keep peripheral vascular resistance at an adequate level, and vasodilators were avoided in the immediate postoperative period. After stabilization, patients were extubated and were shifted to the floor when they were stable. Before discharge from the hospital, we routinely performed transthoracic echocardiography in all patients to determine the adequacy of LVOT obstruction relief and valvular function and to assess for the presence of a VSD or pericardial effusion. Preoperative medications were restarted before patient discharge. We initially prescribed beta-blockers at a lower dose or discontinued entirely. The dosage of these drugs was adjusted in the outpatient clinic during the follow-up.

### Statistical analysis

Statistical analysis was predominantly descriptive due to the size of the cohort. Distributions were described as appropriate by median (interquartile range) or mean ± SD. The analysis was performed using IBM SPSS Statistics software (version 28).

## Results

### Demographic profile and clinical findings of the surgical septal myectomy cohort

During the study period, 29 consecutive patients underwent PlasmaBlade-assisted septal myectomy to relieve symptomatic LVOTO refractory to maximum tolerated medical therapy or as part of other surgical procedures. All the patients were operated by a single surgeon. The mean age of the patients was 60.6 ± 12.5 years, and 17 patients (58.6%) were male. Peak LVOT gradients were 40.5 ± 34.9 mmHg at rest, which increased to 56.5 ± 34.9 mmHg after provocation. Heart failure symptoms consistent with New York Heart Association (NYHA) class III or IV based on functional capacity, including exertional dyspnea and/or chest pain during daily activities, were present in 21 patients (72.4%). Two patients (6.9%) had a history of atrial fibrillation, one patient (3.4%) had first-degree heart block, and one patient (3.4%) had a complete heart block. A total of 15 patients (51.7%) had SAM, and 13 patients (44.8%) had moderate to severe MR. Concomitant severe aortic stenosis was present in five patients (17.2%), and coronary artery disease was present in four patients (13.8%), including one patient (3.4%) who had left anterior descending coronary artery muscle bridge (Table 1). Imaging with two-dimensional echocardiography and/or cardiac CT or CMRI showed anterior ventricular septal thickness in the potential area of muscular resection, measuring 22.1 ± 3.2 mm. The mean left ventricular ejection fraction was 67.9 ± 5.6%, and the ejection fraction was ≥50% in all the patients.

**Table 1:** Demographic profile of the patients operated on for septal myectomy using the PEAK PlasmaBlade.

Age (years)		60.6 ± 12.5
Sex, *N* (%)	Male	17 (58.6%)
	Female	12 (41.4%)
BSA (m^2^)		2.1 ± 0.27
Preoperative rhythm, *N* (%)	Sinus rhythm	17 (58.6%)
	Atrial fibrillation	2 (6.9%)
	First-degree heart block	1 (3.4%)
	Third-degree heart block	1 (3.4%)
Preoperative medications		
	Beta-blockers	22 (75.9%)
	Calcium channel blockers	11 (38%)
Implantable cardioverter defibrillator, *N* (%)		6 (20.7%)
Preoperative echocardiography parameters	LVOT gradient at rest (mmHg)	40.5 ± 34.9
	LVOT gradient after Valsalva (mmHg)	56.5 ± 34.9
	Systolic anterior motion (SAM), *N* (%)	15 (51.7%)
	Mitral regurgitation, *N* (%) Nil	5 (17.2%)
	Mild	11 (37.9%)
	Moderate	10 (34.5%)
	Severe	3 (10.3%)
Additional cardiac lesions, *N* (%)	Severe aortic stenosis	5 (17.2%)
	Coronary artery disease	4 (13.8%)
Functional class, *N* (%)	NYHA I/II	8 (27.6%)
	NYHA III	18 (62.1%)
	NYHA IV	3 (10.3%)
Ventricular septal thickness (mm)		22.1 ± 3.2
Left ventricle ejection fraction (%)		67.9 ± 5.6

BSA, body surface area.

The pre-cardiopulmonary bypass LVOT gradient calculated by direct LV and aortic pressure measurement was 48.1 ± 26.3 mmHg, and it increased to 104.1 ± 49.9 mmHg after induced PVC. All patients underwent septal myectomy, either by the transaortic [27 patients (93.1%)] or transapical [2 patients (6.9%)] approach. Along with septal myectomy, concomitant procedures performed were LAA ligation in 20 patients (69.0%), AVR in 5 patients (17.2%), CABG in 3 patients (10.3%), excision of aortic valve fibroelastoma in 2 patients (6.9%), pulmonary vein isolation in 2 patients (6.9%), left anterior descending coronary artery unbridging in 1 patient (3.4%), and LVOT mass excision in 1 patient (3.4%). Mitral valve intervention for severe mitral regurgitation was required in two patients (mitral valve repair in one patient for a dilated mitral annulus and mitral valve replacement with a bioprosthetic valve in one patient for myxomatous mitral valve with a large anterior mitral leaflet) (Table 2).

**Table 2:** Intraoperative variables in patients operated on for septal myectomy using the PEAK PlasmaBlade.

Pre-CPB LVOT gradient (mmHg)	At rest	48.1 ± 26.3
	After PVC	104.1 ± 49.9
Myectomy, *N* (%)	Transaortic	27 (93.1%)
	Transapical	1 (3.4%)
	Transaortic and apical	1 (3.4%)
Mitral valve surgery, *N* (%)	Accessory chord excision	8 (27.6%)
	Mitral valve repair	1 (3.4%)
	Mitral valve replacement	1 (3.4%)
Additional procedure, *N* (%)	Left atrial appendage closure	20 (69.0%)
	Aortic valve replacement	5 (17.2%)
	Coronary artery bypass grafting	3 (10.3%)
	Other	9 (31.0%)
Aortic cross-clamp time (min)		106.2 ± 62
CPB time (min)		129 ± 69.5
Aortic cross-clamp time for isolated septal myectomy ± LAA closure (min) for the last five cases		55.4 ± 10.7
CPB time for isolated septal myectomy ± LAA closure (min) for the last five cases		73.6 ± 13.4
Post-CPB LVOT gradient (mmHg)	At rest	2.61 ± 2.66
	After PVC	5.48 ± 4.63

### Early postoperative hemodynamics and outcome

No patient had difficulty in weaning from CPB. After myectomy, the resting LVOT gradient by the direct measurement of LV and aortic pressure was reduced to 2.61 ± 2.66 mmHg and increased to 5.48 ± 4.63 mmHg after PVC. All patients had minimal or mild MR. A total of 15 patients (51.7%) patients were discharged on beta-blockers.

No mortality was reported during the index hospitalization. In our early experience, two patients (6.9%) developed ventricular septal defects, measuring 4–5 mm in size. Both defects were identified on post-CPB TEE and were successfully repaired using a bovine pericardial patch. One patient (3.4%) developed CHB, requiring implantation of a permanent dual-chamber pacemaker with ICD. No patient had developed a coronary-cameral fistula on a postoperative echocardiogram. No patient had developed a stroke/neurological deficit in the perioperative period. The mean resting LVOT gradient in the transthoracic echocardiogram performed at discharge was 11.3 ± 9.6 mmHg.

### Histopathological results

**Figure 3:**
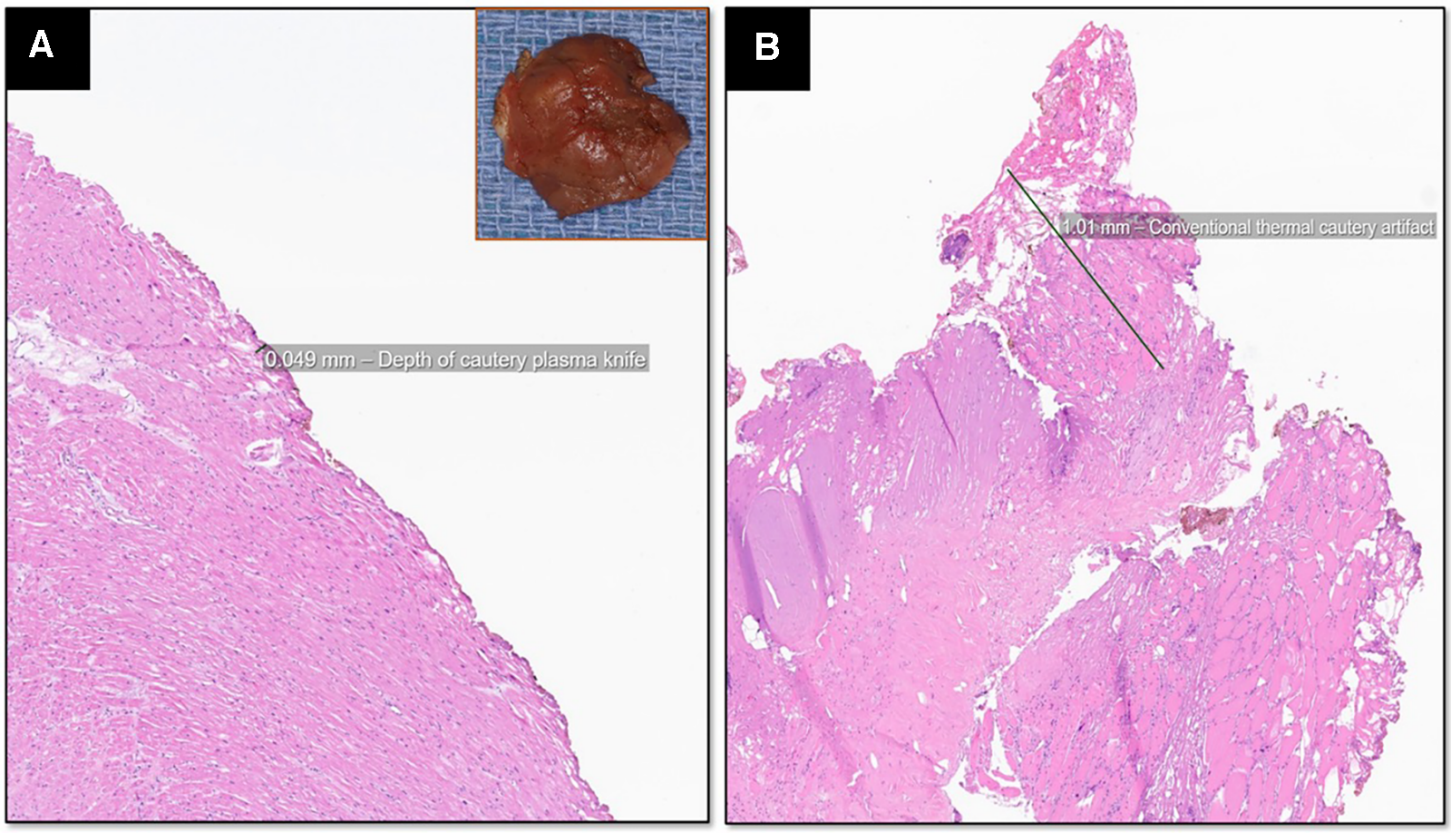
Hematoxylin and eosin, 20× magnification of the myomectomy specimen resected with the PlasmaBlade shows minimal superficial cautery artifact measuring less than 50 µm using digital pathologic measurements (**A**) and the absence of charring on the myectomy specimen (inset). The atrial appendage resected using conventional electrocautery shows 1,000 µm depth of thermal injury that extends from epicardial adipose tissue to cardiac muscle and endothelial vascular lining with loss of normal cellular architecture (**B**).

The myectomy specimen resected using the PlasmaBlade showed minimal superficial cautery artifacts measuring less than 50 µm in depth using digital pathologic measurements (Figure 3A). In contrast, the electrocautery artifact in the LAA specimen was seen to a depth of more than 1,000 µm, and the thermal injury was seen to extend from epicardial adipose tissue to cardiac muscle and endothelial vascular lining (Figure 3B). These results showed that thermal injury spread with the PlasmaBlade was mainly superficial and 20 times less than electrocautery.

### Follow-up

**Figure 4:**
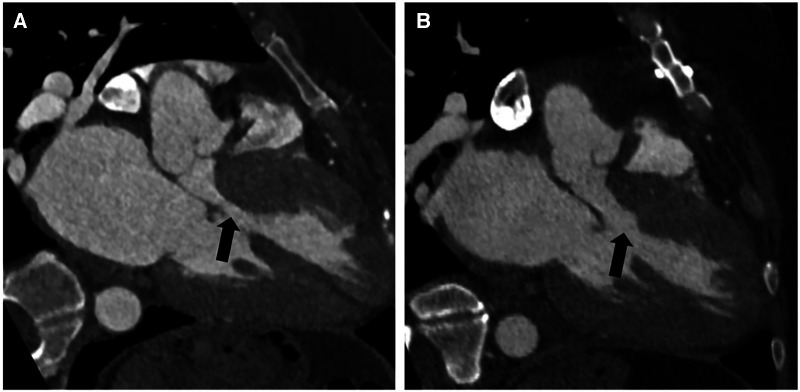
Preoperative contrast-enhanced CT angiography with a three-chamber view during diastole showing asymmetric thickening of the basal septum with narrowing of the left ventricular outflow tract (black arrow) (**A**). Postoperative contrast-enhanced CT angiography with a three-chamber view shows changes from septal myectomy with significantly reduced narrowing of the left ventricular outflow tract compared to preoperative (black arrow) (**B**).

The follow-up was complete, and the mean duration of the follow-up was 8.4 ± 10.3 months. All patients were alive and in NYHA class I. At the last follow-up echocardiography, the mean gradient was 4.4 ± 5.8 mmHg at rest and 9.5 ± 3.3 mmHg after Valsalva. No patient had SAM. There was no coronary-cameral fistula or residual or new flow across the interventricular septum in any patient. No patient required reintervention or reoperation during follow-up (Table 3). Cardiac CECT was performed on two patients for anatomical modeling. As shown in Figure 4, the LVOT is severely obstructed in preoperative CT angiography (Figure 4A). After the septal myectomy using the PlasmaBlade, LVOT is widely open (Figure 4B).

**Table 3:** Postoperative and follow-up data including complications in patients operated on for septal myectomy using the PEAK PlasmaBlade.

Postoperative data		
Complications, *N* (%)	Ventricular septal defect	2 (6.9%)
	Complete heart block	1 (3.4%)
LVOT gradient (mmHg) at time of discharge	At rest	11.3 ± 9.6
	After Valsalva	18.9 ± 15.5
Systolic anterior motion (SAM), *N* (%)		3 (10.3%)
Mitral regurgitation, *N* (%)	Mild	11 (37.9%)
	Moderate	0 (0.0%)
	Severe	0 (0.0%)
Postoperative medications, *N* (%)	Beta-blockers	15 (51.7%)
Follow-up data		
Follow-up duration (months)		8.3 ± 10.4
Complications, *N* (%)		0 (0.0%)
LVOT gradient (mmHg)	At rest	4.4 ± 5.8
	After Valsalva	9.5 ± 3.3

## Discussion

Since 2008, the PlasmaBlade has been in clinical use in Europe and the United States. Significant preclinical and human studies have been conducted with the PlasmaBlade in complex settings, such as otorhinolaryngology, plastic and reconstructive, dermatology, breast, and orthopedic surgery, with excellent results concerning wound healing, damage of surrounding tissue, bleeding control, and inflammation (21). The PlasmaBlade has precision like a knife and hemostasis like electrocautery with little collateral tissue damage, and the same is confirmed by our histopathological results. The use of the PlasmaBlade in cardiac surgery is very limited. A recent study published on PlasmaBlade-assisted internal mammary artery harvesting showed non-inferiority of the PlasmaBlade compared to conventional electrocautery when considering the 6-month patency of the internal mammary arteries (7). Another recently published trial on the safety of using the PlasmaBlade for pocket preparation for ICD device insertion or generator change in patients on anticoagulation has shown significantly reduced adverse events compared to standard electrocautery. Further, there was no reported damage to the lead due to the PlasmaBlade (12, 13).

Precise and adequate resection of hypertrophied muscle without causing damage to the conduction bundle and adjacent valve leaflets is key to the successful outcome of the myectomy procedure. A surgical knife is widely used for muscle resection with good results. A surgical knife results in sharp and precise resection of muscle. Except in the hands of experts, controlling the depth and thickness of muscle excision with a knife is challenging. Sometimes, a large blade and handle may obscure the surgeon's view due to the coaxiality of the knife, and surgeon's visual axis limits the ideal dexterity advantage in tight spaces. This may make the septal muscle excision challenging, especially in small aortic roots, female patients, and narrow spaces. In a recent study published by Juarez-Casso et al. from Mayo Clinic, 1% (27 out of 2,807) patients developed inadvertent aortic valve injury during septal myectomy (6). In a review of the STS database of 1,581 patients undergoing septal myectomy, 1.5% required unplanned AVR (2). However, the mechanism of aortic valve injury is not reported in any study.

In our limited surgical experience, the PlasmaBlade provides the precision of a surgical knife in excising the septal muscle. As the tip of the PlasmaBlade is the only active part, it is easier to control the penetration angle, depth, and thickness of the resection. Further, a plane in previously excised muscle can also be easily created to complete the resection. The long length of the electrode prevents the obstruction of the field of vision. Further, as the blade is active only after pressing the button, the risk of injury to the aortic valve and mitral valve leaflets during insertion or removal through the LVOT is minimal. In our series, we performed transaortic myectomy in 27 patients, transapical myectomy in 1 patient, and transaortic/transapical in 1 patient. The rest of the resection, including incision of hypertrophied and abnormally displaced papillary muscles, resection of accessory LV apical-basal muscle bundles, was also performed using PlasmaBlade. Cutting of the anomalous chordal connections was performed with the help of scissors.

We believe that the very minimal current spread and normal temperature of the PlasmaBlade tip causes minimal collateral tissue damage. This property is essential to prevent necrosis of the healthy myocardium at the site of resection and injury to the conduction bundle. Histopathological examination of our excised myectomy specimens showed that the depth of tissue necrosis was very minimal with the PlasmaBlade (less than 50 µm with the PlasmaBlade vs. more than 1,000 µm with electrosurgical cautery). Our finding is further substantiated by the absence of CHB in all but one patient. Further, no patient developed CHB during the follow-up, suggesting the absence of delayed myocardial necrosis. In cardiac CECT performed during follow-up, no thinning or increased scarring was seen in the ventricular septum at the site of muscle resection. The PlasmaBlade, due to its coagulative property, may also prevent the development of coronary-cameral fistula. No patient in our series reported a coronary-cameral fistula early after surgery or during the follow-up. A coronary-cameral fistula after extended septal myectomy develops due to iatrogenic injury to the intramyocardial branch of the left coronary artery and is reported in 19% and 23% of patients in one retrospective and prospective study, respectively (22, 23). We believe that the absence of a fistula in our series is the result of coagulation of severed intramyocardial branches due to the PlasmaBlade.

Two patients (6.9%) in our first five patients developed VSD, probably due to our learning curve and aggressive resection of the ventricular septum close to the aortic annulus. As our experience with the technique increased, we had no case of VSD in the subsequent 24 cases. The incidence of iatrogenic VSD in our series was significantly higher (6.9%) compared to the reported literature (0.4%–0.7%) (2, 6) due to the smaller number of patients and our learning curve with this technique. In our experience, this technique is easy to learn with a short and steep learning curve of 8–10 cases. Another surgeon in our institution has been able to perform septal myectomy using the PlasmaBlade after observing and cross-scrubbing for five cases. We believe that the use of the PlasmaBlade in other surgical procedures, e.g., pacemaker pocket creation, axillary artery exposure for Impella 5.5 or intra-aortic balloon pump insertion, and pericardiectomy, may increase the comfort level of the surgeon in using the PlasmaBlade and may shorten his learning curve during septal myectomy (24–26). However, our assertion needs further verification with a greater number of surgeons and a larger number of patients. In our series, all patients had nil or single-digit gradient across the LVOT at rest and after Valsalva, immediately after coming off CPB, and the gradient remained low during the follow-up. Widely patent LVOT in CECT chest done in two patients during the follow-up confirms that resection with the PlasmaBlade is durable and does not incite fibrosis. SAM was relieved in all the patients. This shows that adequate and durable muscle resection can be performed with the help of the PlasmaBlade.

### Limitations

Our study has a few limitations. First, it is a retrospective study. Second, the sample size is small, and the follow-up is limited. Third, we did not perform postoperative MRI on any of our patients. We feel that an MRI image may show fibrosis better than a CT scan; however, none of our patients required ablation related to VT storms of abnormal arrhythmias caused by any scar formation, if any. We continue to follow up with our postoperative patients and collect the data for newer patients for the long-term outcome of our technique. In the future, we also plan to compare standard surgical knife-assisted septal myectomy and PlasmaBlade-assisted myectomy. We also recommend larger prospective randomized or non-randomized studies with a greater number of patients and including more surgeons, longer duration of follow-up, and probably cardiac MRI during follow-up to substantiate our findings.

## Conclusion

Adequate surgical septal myectomy can be performed with the help of the PlasmaBlade without the risk of damage to the adjacent myocardium and conduction bundle, with results durable at mid-term. Histologically, the depth of tissue necrosis with the PlasmaBlade is limited to <50 µm thickness with minimal tissue artifact. Larger studies and long-term follow-up data are required to substantiate our results.

## Data Availability

The raw data supporting the conclusions of this article will be made available by the authors, without undue reservation.
